# On the origins of life’s homochirality: Inducing enantiomeric excess with spin-polarized electrons

**DOI:** 10.1073/pnas.2204765119

**Published:** 2022-07-05

**Authors:** S. Furkan Ozturk, Dimitar D. Sasselov

**Affiliations:** ^a^Department of Physics, Harvard University, Cambridge, MA 02138;; ^b^Department of Astronomy, Harvard University, Cambridge, MA 02138

**Keywords:** homochirality, CISS effect, prebiotic chemistry, origin of life, magnetite

## Abstract

Life as we know it is homochiral, but the origins of biological homochirality on early Earth remain elusive. Shallow closed-basin lakes are a plausible prebiotic environment on early Earth, and most are expected to have significant sedimentary magnetite deposits. We hypothesize that ultraviolet (200- to 300-nm) irradiation of magnetite deposits could generate hydrated spin-polarized electrons sufficient to induce enantioselective prebiotic chemistry. Such electrons are potent reducing agents that drive reduction reactions where the spin polarization direction can enantioselectively alter the reaction kinetics. Our estimate of this chiral bias is based on the strong effective spin-orbit coupling observed in the chiral-induced spin selectivity (CISS) effect, as applied to energy differences in reduction reactions for different isomers. In the original CISS experiments, spin-selective electron transmission through a monolayer of double-strand DNA molecules is observed at room temperature—indicating a strong coupling between molecular chirality and electron spin. We propose that the chiral symmetry breaking due to the CISS effect, when applied to reduction chemistry, can induce enantioselective synthesis on the prebiotic Earth and thus facilitate the homochiral assembly of life’s building blocks.

## Significance

Essential biomolecules, like amino acids and sugars, are chiral; they exist in mirror symmetrical pairs named enantiomers. However, modern life selectively uses only one of the enantiomers. The origin of this chiral symmetry breaking remains elusive to date and is a major puzzle in the origin of life research. Here, we consider spin-polarized electrons as potential chiral symmetry-breaking agents utilizing the robust coupling between electron spin and molecular chirality at room temperature as established by the chiral-induced spin selectivity effect. We propose that chiral bias is induced and maintained with enantioselective reduction chemistry driven by such spin-polarized electrons that are ejected from magnetite deposits in shallow prebiotic lakes by solar ultraviolet irradiation.

Chirality is a geometric property, and a molecule that cannot be superimposed on its mirror image is said to be chiral (1). Chiral molecules can be conventionally classified based on their handedness as right-handed (D) or left-handed (L) objects (2). Molecules with opposite handedness (called enantiomers) show identical chemical behavior, although biology is picky when it comes to chirality. Essential molecules for life amino acids and sugars are selectively used in only one handedness. All biological systems predominantly use left-handed amino acids and right-handed sugars. Therefore, homochirality is considered to be a signature of life, and understanding the origins of homochirality is essential for understanding the origins of life. However, the origins of biological homochirality remain an open problem. Nonetheless, it is often acknowledged that its early emergence (e.g., during the prebiotic synthesis of the monomers) would be very advantageous in achieving the efficient polymerization of RNA, which is inhibited in a racemic mixture of nucleotides (3, 4).

Reaching a homochiral state requires at least two things: first, a chiral symmetry-breaking agent that can induce an enantiomeric excess [ee; % ee = 100 × (L –D)/(L + D)] and second, a prebiotically plausible mechanism that can amplify this imbalance (4). Regarding the latter, a number of asymmetric amplification mechanisms have been proposed. Soai and coworkers (5) has reported that asymmetric autocatalysis can generate nearly perfect ee with high yields—although the conditions are not prebiotically plausible. Blackmond and coworkers (6) demonstrated that RNA precursors can be enantioenriched by chiral amino acids in the Powner–Sutherland ribonucleotide synthesis (7). Their scheme showed amplification in the ee of pyrimidine nucleotide precursors, such as glyceraldehyde and aminooxazolines. Blackmond and coworkers (8) also showed that chiral pentose sugars can enantioenrich amino acid precursors with substantial amplification. Their analysis revealed a significant dynamic kinetic resolution that can take advantage of the selective reaction rates for enantiomers and amplify a small ee into near unity. In combination, these results indicate that sugars can trigger the enantioenrichment of amino acids and vice versa, and inducing a small ee can be sufficient to reach an enantiomerically pure state. However, the search for a prebiotically plausible asymmetric amplification mechanism is still active (9). In this work, we are investigating a symmetry-breaking agent that can trigger such an amplification mechanism.

Many chiral symmetry-breaking agents have been proposed—circularly polarized light (CPL), magnetic fields, cosmic rays, and weak nuclear currents to name a few (10–15). These symmetry-breaking agents induce ee by either selectively producing or destroying one isomer more than the other. Studies with CPL take advantage of the optical activity of chiral molecules, and it has been shown that a nearly enantiopure state is reached with the amplification of a slight ee induced by CPL (10, 11, 16). However, the efficiency of CPL is low, and the source and availability of CPL on the early Earth are unclear. Utilizing optical activity of chiral molecules, albeit using unpolarized light in an external magnetic field, Rikken and Raupach (12) demonstrated that an ee of about 100 ppm can be generated. Although this so-called magnetochiral excess is realized with unpolarized light, it relies on the presence of external fields as high as 10 T. Polarized cosmic rays have been theorized as a universal enantioselective agent, inducing a bias during the evolution of two biosystems with opposite handedness (13). Experimental support has come from enantioselective destruction due to dissociative electron attachment (DEA) with low-energy longitudinally polarized electrons by Dreiling and Gay (17), highlighting the viability of longitudinally polarized cosmic beta radiation to do that. Prior experiments by Rosenberg et al. (18) presented a different version of DEA and demonstrated enantioselective destruction of a racemic adsorbed layer of chiral molecules yet with an alternative chiral symmetry-breaking agent: spin-polarized low-energy (a few electronvolts) secondary electrons. The effect they observed is the enantioselective destruction of a chemical bond via high-energy scattering under ultrahigh-vacuum conditions, and it appears to be due to a strong coupling between the spin-polarized electrons and the molecular chiral center (18, 19).

**Fig. 1.**
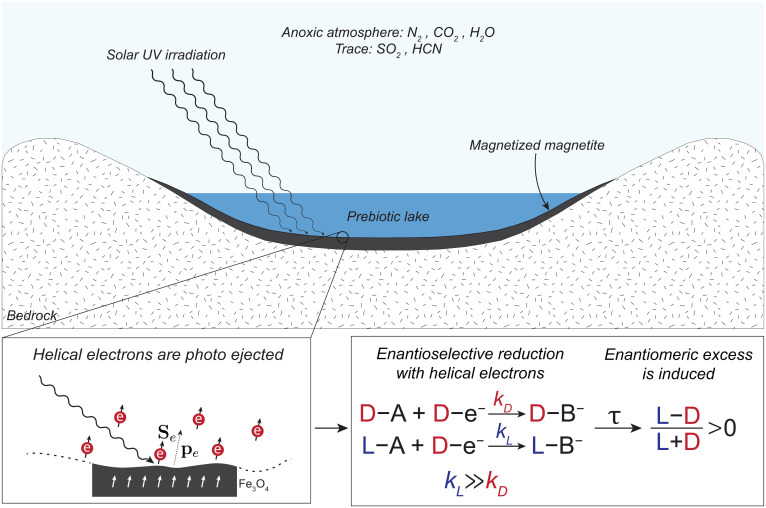
An evaporative lake with magnetite deposits contains the feedstock molecules for prebiotic chemistry. Irradiation of the uniformly magnetized magnetite (Fe_3_O_4_) deposits with solar UV (200- to 300-nm) light generates helical photoelectrons. The helicity of the electrons (D–e–in the figure as the spin and momentum are parallel to each other) is determined by the magnetization direction (section 3 discusses what is meant by the electron helicity). Helical electrons induce CDRC near the magnetite surface due to a selectivity in the reaction rates, *k_L_* and *k_D_*, for different isomers L and D, respectively. This selectivity in the reaction rates can induce an imbalance between two isomers. In the figure, ee in the L isomer is induced.

We suggest that the same strong coupling induces enantioselectivity but with a different source and mechanism. We propose an enantioselective reduction chemistry, in solution, induced by spin-polarized photoelectrons ejected from a magnetized surface, as shown in Fig. 1. The mechanism we suggest is yield preserving as it drives enantioselective production, not destruction. It operates under conditions that are compatible with the conditions of a reduction chemistry at room temperature in solution. The core of our mechanism is the spin–chirality interaction. However, how does spin interact with molecular chirality and induce enantioselective chemistry?

## 1. The Chiral-Induced Spin Selectivity Effect—Chiral Molecules and the Electron Spin

Since the observation of the chiral-induced spin selectivity (CISS) effect, the strong coupling between electron spin and molecular chirality has taken front stage (20). The initial experiments by Naaman and coworkers (21, 22) showed that a self-assembled monolayer of double-strand DNA molecules spin filters a current of photoelectrons on a gold surface. Later studies have confirmed that the effect is induced by the molecular chirality and is robust at room temperature (21, 22). Although a full theoretical framework is still missing, CISS phenomena are qualitatively explained by the coupling of the electron’s linear momentum ($p_{e}$) and spin ($S_{e}$) in the presence of a chiral electrostatic potential $E_{\text{chiral}}$ that changes sign when the handedness is reversed (23). This spin-orbit effect is due to an effective magnetic field, $B=-\frac{1}{c^{2}}v\times E_{\text{chiral}}$, the electron experiences in its rest frame as it goes through a chiral molecule, where **v** is the electron velocity and *c* is the speed of light. The electron interacts with this effective magnetic field through its spin magnetic moment $\mu =-g\mu_{B}S_{e}/\hbar$, where *g* (≈2) is the spin *g* factor, *μ_B_* is the Bohr magneton, and ℏ is the Planck constant. This interaction leads to a splitting in the electron energy as represented by the spin-orbit Hamiltonian $H_{\text{SO}}=-\mu \cdot B$,

[1] $$H_{\text{SO}}=\lambda \hat{n}\cdot \left(S_{e}\times p_{e}\right),$$

where the coefficient *λ* is defined as $\lambda \equiv \frac{E_{0}g\mu_{B}}{\hbar mc^{2}}$ for $E_{\text{chiral}}\equiv E_{0}\hat{n}$ and *m* is the electron mass (23). Therefore, a chiral potential leads to a coupling between the electron’s spin and its linear momentum, and through this coupling, a spin-polarized electron selectively interacts with a chiral molecule.

**Fig. 2.**
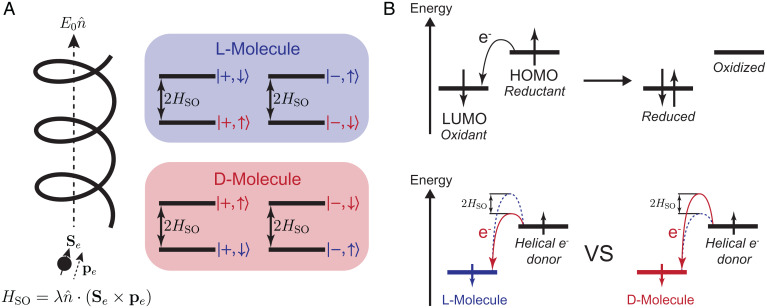
(*A*) The CISS effect strongly couples molecular chirality and electron spin. Electrons interacting with a chiral molecule are spin filtered based on the relationship between their spin and momentum directions. L molecules energetically prefer |D〉 electrons and vice versa, and the energetic difference between two helicity states is given by the effective spin-orbit energy $2H_{\text{SO}}$. (*B*) The driving force of a reduction reaction is the energy difference between the HOMO of the electron donor (reductant) and the LUMO of the electron acceptor (oxidant). The CISS effect causes an energy difference in the activation energies for different isomers when a reduction reaction is driven by helical electrons (right-handed electrons in the figure). An isomer with a lower kinetic barrier (L in the figure) is reduced faster, and this causes enantioselectivity due to the differing reaction rates.

To further elucidate this enantioselective interaction, we can consider the energy splitting associated with the motional and spin states of an electron. With respect to a molecular axis, an electron can move up (+) or down (–), and its spin can be +1/2 (↑) or –1/2 (↓). Therefore, we deal with four states, as shown in Fig. 2*A*: |+,↑〉, |−,↑〉, |+,↓〉, and |−,↓〉. By convention, we define the direction of the chiral potential such that for an electron moving in the + direction through a right-handed molecule, the sign of the magnetic field is positive. Therefore, the interaction of a right-handed molecule with an electron moving in the + direction prefers the –1/2 spin state of the electron. In other words, –1/2 spin state is stabilized as it is lower in energy. Similarly, a left-handed molecule preferably interacts with a +1/2 spin-state electron moving in the + direction. Therefore, we can define doubly degenerate helicity states, |D〉≡{|+,↑〉,|−,↓〉} and |L〉≡{|+,↓〉,|−,↑〉}, for the electron in its interaction with a chiral potential. With this definition, a right-handed molecule energetically prefers |L〉 electron and vice versa, and the penalty for the helicity flip, $P_{\text{flip}}$, is given by the Boltzmann factor corresponding to the energy gap between |L〉 and |D〉 states:

[2] $$P_{\text{flip}}=\exp\left(-\frac{2H_{\text{SO}}}{k_{B}T}\right).$$

This factor is responsible for the spin polarization observed in the CISS experiments as it corresponds to the probability of an electron to be backscattered while retaining its original spin orientation. In order to account for the observed spin polarizations (at room temperature), one needs to consider a spin-orbit energy, $H_{\text{SO}}$, of around 50 meV. For larger chiral molecules, like double-strand DNA, $H_{\text{SO}}$ can be as large as 500 meV, showing that CISS-like effects are robust at room temperature (23). However, we should emphasize that the $H_{\text{SO}}$ we consider and utilize here is not the actual spin-orbit coupling for the molecule but an effective ad hoc value to account for the observations. It is likely that the effective energy, $H_{\text{SO}}$, is due to a combination of spin-orbit and electron spin exchange interactions. The theoretically expected values of spin-orbit coupling for organic molecules are not large enough to explain the observed values of spin polarization. Theory has been catching up recently, and it has been proposed that electron–electron interactions can play a significant role in the CISS effect and explain the high degree of spin polarization for realistic values of spin-orbit coupling (24). Using an intrinsic Rashba coupling approach provides some analogous conclusions (25). However, a unifying theoretical description of all CISS-like effects is still an active area of research (26).

## 2. Enantioselective Reduction Chemistry

The CISS effect demonstrates a strong coupling between molecular chirality and the electron spin that is robust at room temperature. This strong coupling suggests that the CISS effect might also be utilized in reverse; namely, electron spin can bias molecular chirality. In other words, a spin-polarized current of electrons can selectively interact with one handedness as the interaction with the opposite handedness is energetically penalized with $P_{\text{flip}}$. However, how exactly is one handedness differentiated from the other in this process?

Relying on the spin–chirality coupling established by the CISS effect, we propose chiral-induced spin selectivity–driven reduction chemistry (CDRC). This CDRC can induce enantiomeric bias, starting from a racemic mixture, due to the selective reaction rates for different chiralities. Consider two reduction reactions, in a solution, that are identical except that one is for an L molecule (L–A) and the other is for a D molecule (D–A):

$$(\text{L/D})-{\text{A}+\text{e}}^{-}\to (\text{L/D})-{\text{B}}^{-}.$$

**Fig. 3.**
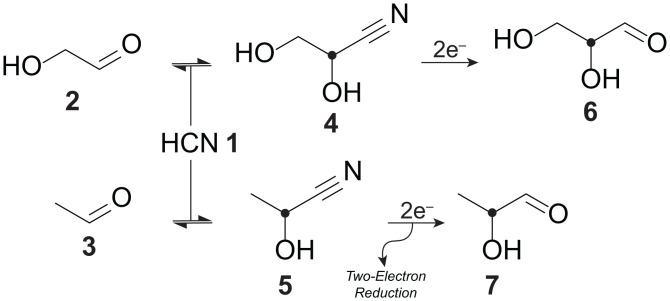
The cyanosulfidic prebiotic chemistry uses solvated electrons as the main reducing agent. The enantioselective reduction scheme we propose can be applied to the cyanosulfidic chemistry when a chiral center is produced and is subjected to a reduction by helical electrons. Black dots show the chiral centers. Adapted from ref. 46, which is licensed under CC BY 4.0.

When the reduction is driven by nonpolarized electrons, e–, the rates of two reactions are on average identical, and the L molecule yields an L product (L–B–) and vice versa with the same rate. This is because, on average, half of the electrons are in the right-handed helicity state, and they react faster with left-handed molecules; the other half of the electrons are in the left-handed helicity state, and they react faster with the right-handed molecules. Therefore, the balance is preserved. However, if the helicity of electrons is biased toward one direction, then the enantiomeric selection occurs as one handedness would on average react faster than the other. The difference in the reaction rates is due to the Arrhenius relation for which the reaction rate, *k*, is proportional to an exponential factor decreasing with higher activation energy: $\exp\left[-\frac{\left(E_{a}\pm H_{\text{SO}}\right)}{k_{B}T}\right]$, where *E_a_* is the bare activation energy without the CISS correction. Therefore, the Arrhenius relation predicts a faster reaction rate by $\exp\left(\frac{2H_{\text{SO}}}{k_{B}T}\right)$ for the “opposite” molecular chirality. For example, for a reduction reaction driven by electrons in the right-handed helicity state, D–e–, we claim that the left-handed reagent, L–A, reacts faster:

$$\begin{array}{l} \text{D}-\text{A}+\text{D}-{\text{e}}^{-}\to \text{D}-{\text{B}}^{-}:\;k_{D}\\ \text{L}-\text{A}+\text{D}-{\text{e}}^{-}\to \text{L}-{\text{B}}^{-}:\;k_{L}, \end{array}$$

where $\frac{k_{L}}{k_{D}}=\exp\left(\frac{2H_{\text{SO}}}{k_{B}T}\right)$ due to the proposed CDRC. This ratio between the reaction rates for different enantiomers varies from five to several thousand for effective spin-orbit energies (20−100 meV) typically used to account for CISS measurements (23). Above, we considered the reduction of a chiral molecule, not the formation of a new chiral center from an achiral precursor (e.g., reduction of **4** to **6** in Fig. 3). However, the same effect can still play a role in the latter as soon as there is a chiral reaction intermediate involved in the electron exchange.

For a microscopic explanation of the enantioselectivity in reduction chemistry, we can look into the molecular orbital energies of molecules involved in the electron exchange. Let us first consider a regular redox reaction with nonchiral molecules where the electrons are flowing from a reductant to an oxidant as in Fig. 2*B*. The driving force for this electron transfer is the energy difference between the highest occupied molecular orbital (HOMO) of the reductant and the lowest unoccupied molecular orbital (LUMO) of the oxidant, and this positive energy difference makes the electron transfer thermodynamically favorable (downhill). However, when the electron acceptor (oxidant) is a chiral molecule and the electron donor (reductant) is ejecting helical electrons, then the activation energy of the reaction will depend on the molecular handedness. The energy difference between the activation energies for L and D molecules ($2H_{\text{SO}}$) causes a difference between the reaction rates for two isomers, and it is the reason behind the enantioselectivity of the reduction reaction. Therefore, the CISS effect breaks the chiral symmetry of the reduction reaction as it alters the reaction kinetics for enantiomers. We can see a manifestation of this in the recent electrochemistry experiments with a magnetized electrode, where the electron spin direction enantioselectively affects the redox behavior of a chiral ferrocene derivative, Ugi’s amine, and camphorsulfonic acid (27, 28). Similarly, it has been shown that chiral centers can be enantioselectively formed by a solid-state catalytic reaction on a magnetized hematite (Fe_2_O_3_) surface due to enantioselective reaction kinetics by the CISS effect (29).

We must note that spin polarization of electrons per se is not enough for enabling CDRC. This is because the CISS effect requires a well-defined relationship between the spin and momentum vectors. In fact, CISS is the filtering of longitudinally polarized electrons where spin and momentum vectors are colinear (23). Hence, one cannot realize CISS-like effects with spin-polarized electrons with arbitrary momenta. Thermally polarized electrons under very strong magnetic fields or spin-polarized electrons attained by the Triplet Mechanism (30, 31) are examples where electronic spin polarization is achieved but helicity is not. This brings up the question of where one could find spin-polarized electrons with well-defined momenta in a prebiotic setting.

## 3. Prebiotic Environments, Evaporative Lakes, and Magnetite Formation

The irradiation of a ferromagnetic substrate can provide secondary spin-polarized electrons with an aligned flux of momenta. Due to angular momentum conservation, the spin of electrons ejected from a magnetic surface are aligned. Moreover, the backscattered photoelectrons move away from the surface normal, $\hat{n}$, and thus, have aligned momenta with respect to the surface: $\langle \hat{n}\cdot p_{e}\rangle \neq 0$, where brackets stand for an average over the ensemble of electrons. Therefore, the spin and momentum vectors of these electrons have a well-defined relationship with respect to each other, and these electrons are said to have a helical character. This helicity is on average positive (negative), $\langle S_{e}\cdot p_{e}\rangle >0$ (<0), if the surface is magnetized upward (downward) and the electrons are said to be right handed (left handed) by convention. Hence, electrons ejected from a magnetized surface are helical electrons, and their helicity changes depending on the magnetization direction of the substrate. In Fig. 1, right-handed helical electrons are illustrated for which the overlap of spin and momentum vectors is positive.

Such magnetized surfaces—namely of magnetite—might have been common on prebiotic early Earth (and early Mars) in the basins of closed evaporative lakes, which also provide environments for surficial origins of life scenarios (32–34). Under anoxic conditions and lake waters rich in dissolved iron, the redox-stratified water column will allow the accumulation of iron oxides deposits—primarily magnetite (Fe_3_O_4_) in the deeper layers below the ultraviolet (UV)-light photic zone (35–37). Evidence for an ancient redox-stratified lake with underlying magnetite-rich sediment was uncovered by the Curiosity rover in the Gale crater on Mars (38). Gale Crater Lake seems to be a good analog for aqueous basins that evolve into environments for origins of life chemistry (figure 2 in ref. 33 and the discussion of authigenic magnetite formation). Note that magnetite formation in such lakes would normally precede—by $10^{3}$ to $10^{5}$ y—the prebiotic chemistry pond (figure 2A vs. figure 2D in ref. 33), and hence, the flat magnetite surfaces can be exposed to UV light in the shallow photic zone at that later time (Fig. 1).

Magnetite has a particularly well-matching work function of around 4 eV (310 nm) (39, 40) to the anoxic (no ozone) atmosphere of early Earth, and the irradiation of a natural magnetite near its photothreshold has been shown to generate spin-polarized electrons with around –40% polarization (40, 41). The low value of the work function of magnetite allows for the generation of photoelectrons for the UV wavelengths below 310 nm that were abundant (around two orders of magnitude higher compared with now) on the surface of a prebiotic lake (42). Magnetite is also the most common and naturally magnetic mineral on Earth, with a saturation magnetization of 480 kA/m (6,000 G) and a Curie temperature of $580{\;}^{{}^{\circ}}$C. Moreover, magnetite maintains magnetization after the external field is removed and the mineral is thermally processed, having a thermoremanent magnetization of 4.8 to 7.2 kA/m (60 to 90 G) (43). Therefore, within the mesoscopic vicinity of the magnetite deposits, there exists a magnetic field stronger than that of the Earth’s (around 0.5-G) field. Individual magnetite sediments in a large area (continent size to hemisphere) will share the same field orientation. Hence, this uniform and relatively strong field can open up the possibility of having magnetic field effects in the aqueous chemistry through spin effects—especially in regard to photochemical reactions (44, 45).

## 4. Relevance to Prebiotic Chemistry

We suggest that CDRC with spin-polarized electrons can be realized in prebiotic chemistry networks and that an ee can be induced right after the generation of the first chiral molecules. The cyanosulfidic chemistry is an especially attractive candidate as it utilizes solvated electrons for the synthesis of biomolecules, including the first chiral sugar and chiral precursors of amino acids (46, 47). Furthermore, the chemistry is a surface pond chemistry where high-energy UV photons are available for the generation of spin-polarized photoelectrons from a magnetized surface on the shallow lake bed.

We assume that UV-generated photoelectrons from the magnetite will solvate in water and be utilized effectively in the cyanosulfidic chemistry. Such solvation was demonstrated recently in experiments where photoelectrons ejected from a metallic surface upon UV irradiation became hydrated at the metal–liquid interface (48). In the cyanosulfidic chemistry, hydrated electrons are generated from the photoredox cycling of dissolved ferrocyanide and used in the reductive homologation of hydrogen cyanide—the main feedstock molecule (32, 47). The electrons generated in the bulk by photoredox cycling do not have a helical character; therefore, they do not react enantioselectively. However, we suggest that helical electrons ejected from the magnetite surfaces with a long spin decay time of 8  μs (49) can induce enantioselective reduction reactions.

As illustrated in Fig. 3, during the first reduction stage, glycolaldehyde **2** and acetaldehyde **3** are synthesized from glycolonitrile. Following this step, glycolaldehyde cyanohydrin **4** and acetaldehyde cyanohydrin **5** are yielded by a Killiani–Fischer-type growth of glycolaldehyde and acetaldehyde with hydrogen cyanide **1**, respectively (46). These cyanohydrins are the first chiral molecules in the cyanosulfidic chemistry, and they undergo a prompt reduction with solvated electrons to produce glyceraldehyde **6** (first chiral sugar) and lactaldehyde **7** (threonine precursor). This is the step where CDRC can play a role as we consider the reaction of a chiral molecule with an electron. If electrons with defined helicity are used to induce this reduction of chiral cyanohydrins, we suggest that an ee can be induced in the synthesis of glyceraldehyde and amino acid precursors.

Given that glyceraldehyde **6** is also shown to play a vital role in the Powner–Sutherland-type synthesis of nucleotides (7), inducing an ee at the early stages of the prebiotic chemistry network is significant, as it carries on at the later stages. Having a persistent enantiomeric bias increases the yield of each step, and this becomes especially important in such multistep reaction networks where the overall yield can be quickly converged to zero by the so-called arithmetic demon (50, 51).

In addition, the effects of photooxidation on the magnetite surface can be reversed, and the surface can be refreshed by reductants such as bisulfite (HSO_3_^−^), which has also been shown to concentrate and stabilize aldehydes and prevent the racemization of glyceraldehyde into dihydroxyacetone (52).

Note that the enantioselective effect we propose can be considered in combination with a dynamical kinetic resolution scheme that can amplify induced ee. If helical electrons can indeed lead to a selectivity in the reaction rates for different enantiomers at the reduction stage (e.g., **4** to **6**), dynamic resolution can enhance the ee at the product stage (6) to more than 50% due to racemization (2⇌4) of the reagent (4).

## 5. Discussion

Let us summarize the features of CDRC as a potential mechanism for inducing ee in prebiotic chemistry and therefore, being the trigger of biological homochirality.

The basic mechanism behind CDRC is the robust coupling between molecular chirality and the electron spin established by the CISS effect. As such, CDRC is a robust and deterministic way of inducing ee at room temperature and in solution.

Moreover, the mechanism we suggest is prebiotically plausible. Helical photoelectrons can be generated by irradiating magnetized magnetite deposits with UV light. Therefore, the chiral symmetry-breaking agents—the helical electrons—are generated in situ with the biomolecules, and their supply is robust and long lasting. Magnetite minerals and high-energy UV radiation capable of ejecting photoelectrons are expected to be available in prebiotic lake environments.

CDRC takes advantage of the exponential decrease in the reaction rates with the activation energy. Therefore, even for small chiral molecules with effective spin-orbit couplings of around 50 meV, it is possible to achieve a 50-fold difference in the reaction rates for the two enantiomers. Furthermore, our mechanism induces ee by means of synthesis, not by enantioselective destruction. Therefore, it does not deteriorate the reaction yields in a multistep reaction network. This adds to the increasing reaction yields in the approach to a enantiomerically purer mixture and boosts the prebiotic plausibility of the entire network.

The fundamental coupling at the core of the CDRC is compatible with any chiral molecule as long as it reacts with low-energy spin-polarized electrons. Hence, it would apply to various reaction steps where a chiral molecule is reduced in any prebiotic chemistry scenario. For a network, of course, the earlier the better, as it will contribute to higher yields.

As prebiotic chemistry scenarios go, CDRC seems best applicable to the cyanosulfidic chemistry network (32, 47) because UV-generated solvated electrons are essential in the initial synthesis. Starting with the second-stage reduction of nitriles in the cyanosulfidic network, one can utilize helical photoelectrons to induce ee on (or near) the surface, while nonhelical solvated electrons in the bulk still react in a nonenantioselective way. Therefore, in the prebiotic scenario we conceive, surface electrons induce ee via CDRC, and the bulk electrons, generated by photoredox cycling, keep acting as the main reducing agent. Although the nonhelical bulk electrons are likely to outnumber the surface electrons due to the low efficiency [$10^{-7}$ to $10^{-2}$ depending on the photon energy (39)] of backscattering in the photoelectric effect, ee can still be induced near the surface. However, because we do not yet know the yield-limiting step of the whole process, it is hard to accurately estimate the helical solvated electron yield and the subsequent efficiency of inducing ee. We, therefore, intend to address the yields question with future experimental work.

Another remaining question is if the helical electrons will remain helical when they are solvated. This is an important step because when photoelectrons are ejected from the magnetized surface, they have a finite length scale before their helicity is lost. Although it is hard to predict the hydration dynamics of a spin-polarized electron and estimate the lifetime of the helical electrons, the long spin-relaxation time of solvated electrons [T_1_ ≈8  μs (49)] and the observation of the spin-dependent electrochemical behavior with magnetized electrodes in solution are encouraging (27, 28). We hope that future experiments under study are going to elucidate the validity of the aforementioned claims.

### 5.1. Proposed Experiments.

We conceive of several experiments to test the validity of the CDRC and its proposed applicability to the cyanosulfidic chemistry.

Electrochemistry is a well-suited platform to study electron exchange reactions near an electrode surface. We propose studying the reduction reactions in cyanosulfidic chemistry (e.g., **4** to **6**) in an electrochemical cell with a magnetized working electrode at a fixed reductive potential (27, 28). Magnetized electrodes can provide helical electrons into a solution of racemic glycolaldehyde cyanohydrin **4** and acetaldehyde cyanohydrin **5**, and the proposed enantioselective reduction of these species can be tested. The reduction products of these cyanohydrins, glyceraldehyde and lactaldehyde, have relatively high UV absorption in the 250- to 300-nm range, and therefore, their ee can be monitored via circular dichroism (CD) spectroscopy in this wavelength range. Monitoring the induced ee as a function of reaction time on a CD spectrometer can also reveal the kinetic properties of the proposed enantioselective reduction.

Similar to the experiment proposed above, one can prepare a racemic mixture of **4** and **5** in solution on a magnetized magnetite surface and expose this solution to UV light (λ<310 nm), where helical electrons can be photoejected into the solution. Using similar analytical methods proposed for the electrochemical reduction near the magnetized surface, one can monitor if helical photoelectrons induce ee or not.

Moreover, magnetic-conductive atomic force microscopy (mc-AFM) measurements can reveal the spin-polarization properties of the molecules of interest of prebiotic chemistry due to the CISS effect (53–55). In an mc-AFM experiment, a spin-polarized current of electrons is transmitted from a magnetic surface to the atomic force microscopy tip via a monolayer of chiral molecules on the surface. Due to the CISS effect, the magnitude of the transferred current depends on the spin-polarization direction with respect to the chiral molecular axis. Therefore, we can conceive of directly probing the CISS properties of the chiral molecules in the cyanosulfidic chemistry with mc-AFM measurements and quantitatively test the CISS effect origins of the CDRC idea.

## 6. Conclusion

This work offers a prebiotically plausible mechanism for elucidating the origin of life’s homochirality. We propose spin-polarized electrons ejected from magnetized magnetite deposits by UV irradiation as likely candidates for breaking the chiral symmetry in prebiotic chemistry. The suggested mechanism relies on the interaction between electron spin and molecular chirality—whose magnitude is empirically derived from the CISS effect—and it anticipates different synthetic reaction rates for the enantiomers. Thereby, the reduction with helical electrons can break the chiral symmetry and pave the way for enriching the ee over time, where the enhancement rate is determined by the difference in the reaction rates for specific enantiomers. The mechanism is robust at room temperature in solution and is applicable to any chiral structure. It offers an in situ and continuous generation of ee in a prebiotic lake environment. Moreover, the generation of ee with spin-polarized electrons seems well suited to surficial prebiotic chemistry scenarios, like the cyanosulfidic chemistry, which relies on solvated secondary electrons as its main reducing agent. We expect that future experiments can test the mechanism’s viability.

## Data Availability

There are no data underlying this work.
